# In Vitro Evaluation of Lipopolyplexes for Gene Transfection: Comparing 2D, 3D and Microdroplet-Enabled Cell Culture

**DOI:** 10.3390/molecules25143277

**Published:** 2020-07-18

**Authors:** Juan L. Paris, Filipe Coelho, Alexandra Teixeira, Lorena Diéguez, Bruno F. B. Silva, Sara Abalde-Cela

**Affiliations:** International Iberian Nanotechnology Laboratory (INL), Avda Mestre José Veiga, 4715-310 Braga, Portugal; filipe.coelho@inl.int (F.C.); alexandra.teixeira@inl.int (A.T.); lorena.dieguez@inl.int (L.D.)

**Keywords:** Lipopolyplex, gene transfection, multicellular tumor spheroid, single-cell microdroplets, in vitro evaluation

## Abstract

Complexes combining nucleic acids with lipids and polymers (lipopolyplexes) show great promise for gene therapy since they enable compositional, physical and functional versatility to be optimized for therapeutic efficiency. When developing lipopolyplexes for gene delivery, one of the first evaluations performed is an in vitro transfection efficiency experiment. Many different in vitro models can be used, and the effect of the model on the experiment outcome has not been thoroughly studied. The objective of this work was to compare the insights obtained from three different in vitro models, as well as the potential limitations associated with each of them. We have prepared a series of lipopolyplex formulations with three different cationic polymers (poly-l-lysine, bioreducible poly-l-lysine and polyethyleneimine), and assessed their in vitro biological performance in 2D monolayer cell culture, 3D spheroid culture and microdroplet-based single-cell culture. Lipopolyplexes from different polymers presented varying degrees of transfection efficiency in all models. The best-performing formulation in 2D culture was the polyethyleneimine lipopolyplex, while lipoplexes prepared with bioreducible poly-l-lysine were the only ones achieving any transfection in microdroplet-enabled cell culture. None of the prepared formulations achieved significant gene transfection in 3D culture. All of the prepared formulations were well tolerated by cells in 2D culture, while at least one formulation (poly-l-lysine polyplex) delayed 3D spheroid growth. These results highlight the need for selecting the appropriate in vitro model depending on the intended application.

## 1. Introduction

The introduction of nucleic acids in target cells to treat diseases (gene therapy [1]) holds great promise in many pathological scenarios [2,3,4,5,6,7]. Viral vectors have been thoroughly studied for this purpose, but concerns about their safety profile, poor scalability and high costs lead researchers to look for alternative nonviral gene delivery agents [8]. Many types of synthetic or natural structures have been studied to carry the desired nucleic acids (DNA or RNA) inside target cells, with varying degrees of success [9,10,11,12]. Among the most promising types of carriers for gene delivery are complexes of lipids, polymers and nucleic acids (lipopolyplexes (LPPs)) [13]. To obtain LPPs, a cationic polymer is commonly used first to form a complex with the negatively charged nucleic acid, forming a polyplex (PP). This PP can be later coated with a liposome to obtain the final LPP structure [14]. LPPs provide great versatility, since a wide range of different lipids, polymers and nucleic acids can be modularly selected to adapt the formulation to the particular needs of many pathological scenarios. Thus, they have been used for producing nanovaccines [15], for the treatment of neurodegenerative diseases [8], and especially for cancer treatment [16,17,18,19,20,21,22].

**Figure 1:**
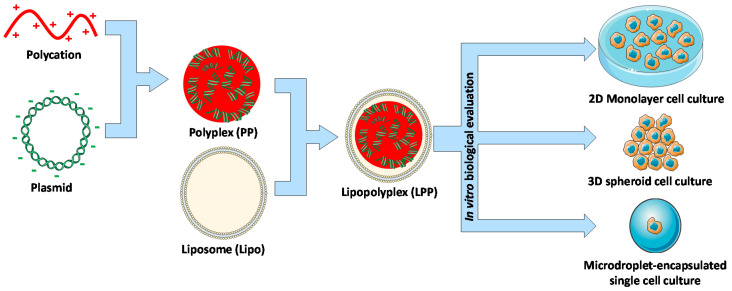
Schematic representation of the work carried out.

When a novel gene delivery formulation is prepared, one of the first steps to test their therapeutic potential is to evaluate their gene transfection capacity in vitro [23]. In this first evaluation, some promising compositions might be selected to be further modified, or to be tested in a more complex biological model, such as in vivo evaluation. However, a multitude of different in vitro culture models are available, and although there have been attempts to standardize this evaluation [24], the effect of the model selected on the formulation performance has not been thoroughly evaluated for most types of formulations. In addition, the suitability of the in vitro culture model is certainly dependent on the disease that the formulation is intended for. While early-stage investigations for more general formulations should not be discouraged from using simplistic 2D cell cultures, it is certainly important to understand the virtues and drawbacks of each method. The objective of this work was to evaluate a series of LPP formulations delivering a reporter plasmid through three different in vitro culture models, to understand the effect of the selected model on their biological performance (Figure 1). Along with this evaluation, we also characterize the negative PP component of the LPP as a control.

The formulations were prepared employing three different cationic polymers: poly-l-lysine (PLL), bioreducible poly-l-lysine (bPLL) and polyethyleneimine (PEI). PLL has been widely studied to complex nucleic acids, although its transfection capacity is generally limited, so degradable versions such as bPLL have been proposed to improve it [25]. bPLL is obtained by introducing disulfide bonds within its polymeric chain that are cleaved when exposed to the reducing intracellular environment [25,26]. PEI is one of the most widely used components in nonviral gene delivery vectors due to its capacity to capture and release nucleic acids in a biological setting, as well as by its enhanced endosomal escape capacity (due to the proton sponge effect) [27,28,29,30]. These polymers were incubated with a green fluorescent protein (GFP) plasmid to obtain PPs. Subsequently, these PPs were combined with cationic liposomes (Lipos) to obtain LPPs. The prepared PPs and LPPs were evaluated through the following in vitro models with A549 adenocarcinomic human alveolar basal epithelial cells [31,32,33]: 2D monolayer cell culture, 3D multicellular spheroid culture and microdroplet-based single-cell culture. Two-dimensional (2D) monolayer culture is the easiest and most commonly used model to evaluate formulations for drug and gene delivery [23,30,34]. However, the main limitation of this model is that the cells grow in a completely different conformation than they naturally would in vivo, which is known to alter their metabolism and their response to different stimuli [35]. Furthermore, 2D monolayer culture does not mimic the natural structure and interactions (cell–cell and cell–extracellular environment) that would be present in real tissue. To attenuate these limitations, 3D multicellular spheroid cultures have been proposed as a more representative model of what might happen in a solid tumor scenario [35,36,37], showing for example a hypoxic region in their center [38,39]. In this model, cells grow as a mass of cells not attached to the bottom of the well plate, presenting some of the biological barriers that would be displayed in vivo, such as the need for tissue penetration to reach most of the target cells. Finally, a microdroplet-based single-cell culture was evaluated as a model to gain mechanistic insights on the transfection process [40]. On the one hand, single-cell analysis could reveal novel insights regarding delivery and transfection kinetics [41], for example as a function of individual cell phenotypic characteristics. On the other hand, being a microfluidics-based platform could also enable high-throughput analysis [42]. In this model, single cells were encapsulated in microdroplets together with the formulations, enabling a strong interaction between them while avoiding interactions with other surrounding cells. Furthermore, inside these microdroplets the cells are suspended exposing all of their surfaces to the medium with the formulation, unlike what would happen in 2D monolayer culture, where only the nonattached surface is available for interaction with the particles.

## 2. Results and Discussion

### 2.1. Particle Synthesis and Characterization

PPs were prepared by mixing a plasmid encoding for GFP and different cationic polymers: PLL (PP PLL), bPLL (PP bPLL) and PEI (PP PEI). Subsequently, Lipos labeled with Texas Red were added to each of these PP formulations to obtain the corresponding LPPs. The prepared particles were characterized by dynamic light scattering (DLS) and Z Potential (Table 1).

Z Potential results show that the obtained PPs presented a negative surface charge, as was desired, to be able to coat them with Lipos (which showed high positive surface charge). After the addition of Lipos, the surface charge of all of the LPP formulations increased when compared to the original PP samples, becoming positive for LPP PLL and LPP PEI, and closer to neutral for LPP bPLL. Average particle sizes varied in the range between 87 and 168 nm, suitable for their subsequent in vitro biological evaluation as potential nonviral gene delivery vectors [16]. Finally, the core@shell structure of the LPPs proposed in Figure 1 was confirmed by Transmission Electron Microscopy (TEM) with negative staining (representative micrographs from PP PLL, Lipo and LPP PLL can be found in Figure S1).

### 2.2. Biological Evaluation in A 2D Monolayer Cell Culture Model

First, a 2D monolayer cell culture was employed to test the transfection efficiency of the prepared structures, as this is the most common model employed in the early development of gene delivery agents. For this, A549 cells were seeded in well plates 24 h prior to their incubation with the particles. Three days after particle addition, particle uptake and transfection efficiency were evaluated by fluorescence microscopy and flow cytometry.

**Figure 2:**
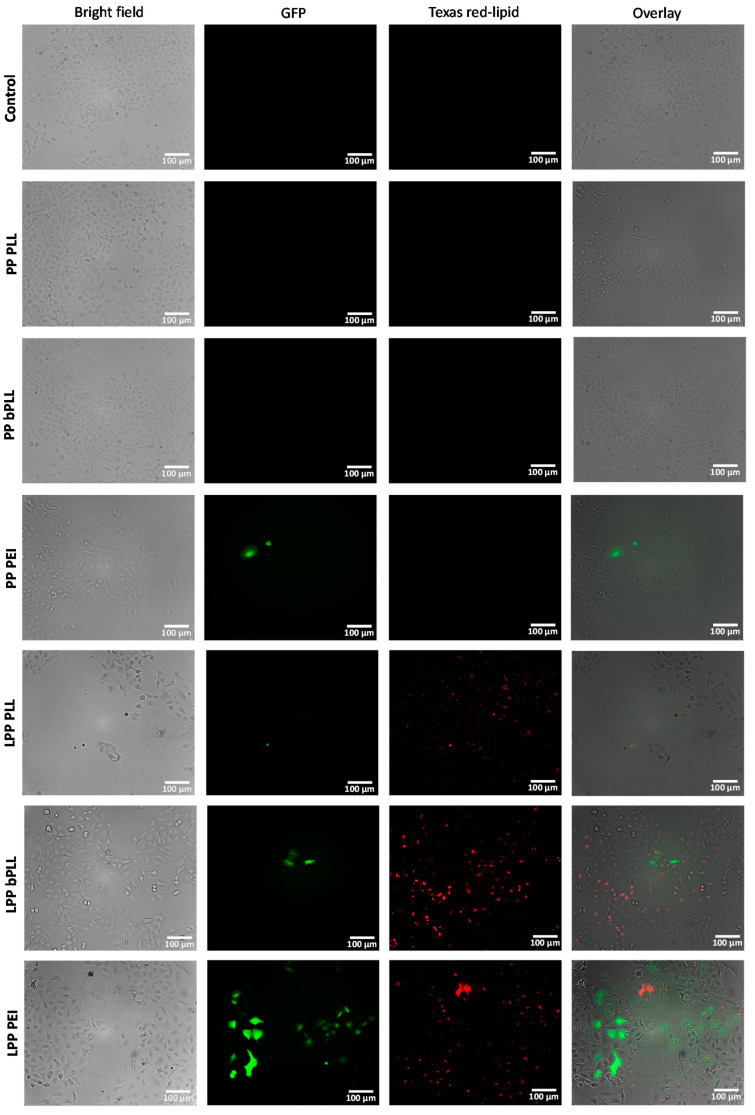
Fluorescence microscopy images of A549 cells in 2D monolayer cell culture 3 d after incubation with polyplexes (PP) and lipopolyplexes (LPPs). The figure shows, from left to right, for each treatment: bright-field image, green fluorescent protein (GFP) fluorescence (green), Texas Red lipid fluorescence (red) and overlay of the three previous images.

The results obtained by fluorescence microscopy (Figure 2) show that uptake of all LPP formulations appeared to be effective, since red fluorescence from a labeled lipid in the formulation was observed in most of the cells in the culture. The uptake of PP formulations could not be evaluated in this way, since the formulation lacked a fluorescent component. From the images, GFP transfection seemed efficient for LPP PEI and only slightly effective for LPP bPLL, while virtually no transfection was seen in any of the other conditions. This result was in good agreement with previous reports that indicated a limited transfection efficiency of gene delivery vectors based on PLL, which could be improved by developing systems of bPLL [25], or employing PEI as an alternative [14]. The lack of transfection capacity of the PP formulations can also be explained by the low charge ratio (CR) used, providing clearly negatively charged particles that are known to be poor gene delivery vectors [23].

**Figure 3:**
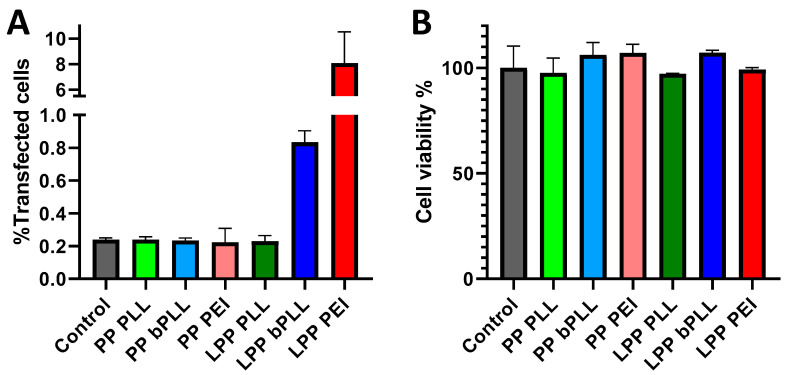
Flow cytometry evaluation of A549 cell transfection in 2D monolayer cell culture 3 d after incubation with PPs and LPPs (**A**); Resazurin viability assay of A549 cells incubation with PPs and LPPs for 24 h (**B**). Data are Means ± SD, *n* = 3.

These results were further confirmed by quantitative analysis of cell fluorescence performed by flow cytometry (Figure 3A). The measured percentage of transfected cells with LPP PEI (ca. 8%) was in a similar range as what was recently described for other PEI-based nonviral gene delivery systems evaluated in A549 cells [43], although similar systems have reported values up to 30% [29,44]. The cytotoxic potential of the prepared PPs and LPPs was also evaluated in this 2D monolayer culture model by means of a resazurin-based assay (Figure 3B and Figure S2). No signs of toxicity were detected for any of the prepared formulations after 24 h or 72 h of continuous contact with the cells.

### 2.3. Biological Evaluation in A 3D Multicellular Spheroid Culture Model

**Figure 4:**
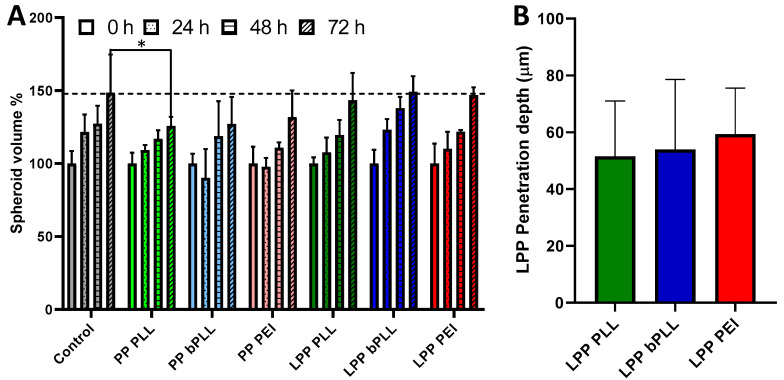
Growth of A549 multicellular spheroids treated with PPs and LPPs for 3 d (**A**); Penetration depth of LPPs (with Texas Red-labeled lipid) in 3D spheroids evaluated by confocal microscopy (**B**). Data are Means ± SD, *n* = 3. * *p* < 0.05 (Student’s t-test). The average initial spheroid size was 507.2 ± 19.5 µm.

The second in vitro model tested consisted of 3D multicellular spheroids. A549 tumor spheroids were prepared using nonadherent round-bottom culture well plates one week before the addition of nanoparticles. Then, the different PP and LPP formulations were incubated with the spheroids for three days, evaluating their size along this period of time, as well as carrying out confocal microscopy on the last day. At the beginning of the experiment (on the day that the formulations were added), the spheroids had an average diameter of 507.2 ± 19.5 µm. Significant growth was observed in all of the experimental groups when compared to the initial spheroid volume, up to 50% increase during the three days of the experiment (Figure 4A). While by the end of the experiment the spheroids incubated with LPPs showed an almost identical volume when compared to control spheroids, PP-treated spheroids presented an average volume smaller than the control, although the difference was only statistically significant for the spheroids incubated with PP PLL. This result indicates that the formulation PP PLL hinders the growth of A549 spheroids, indicating some negative effect on A549 cell viability or proliferation that was not apparent in the 2D cytotoxicity experiment previously performed. This highlights the fact that the selection of in vitro models for drug testing can affect the obtained results, even when apparently analogous results would be expected.

**Figure 5:**
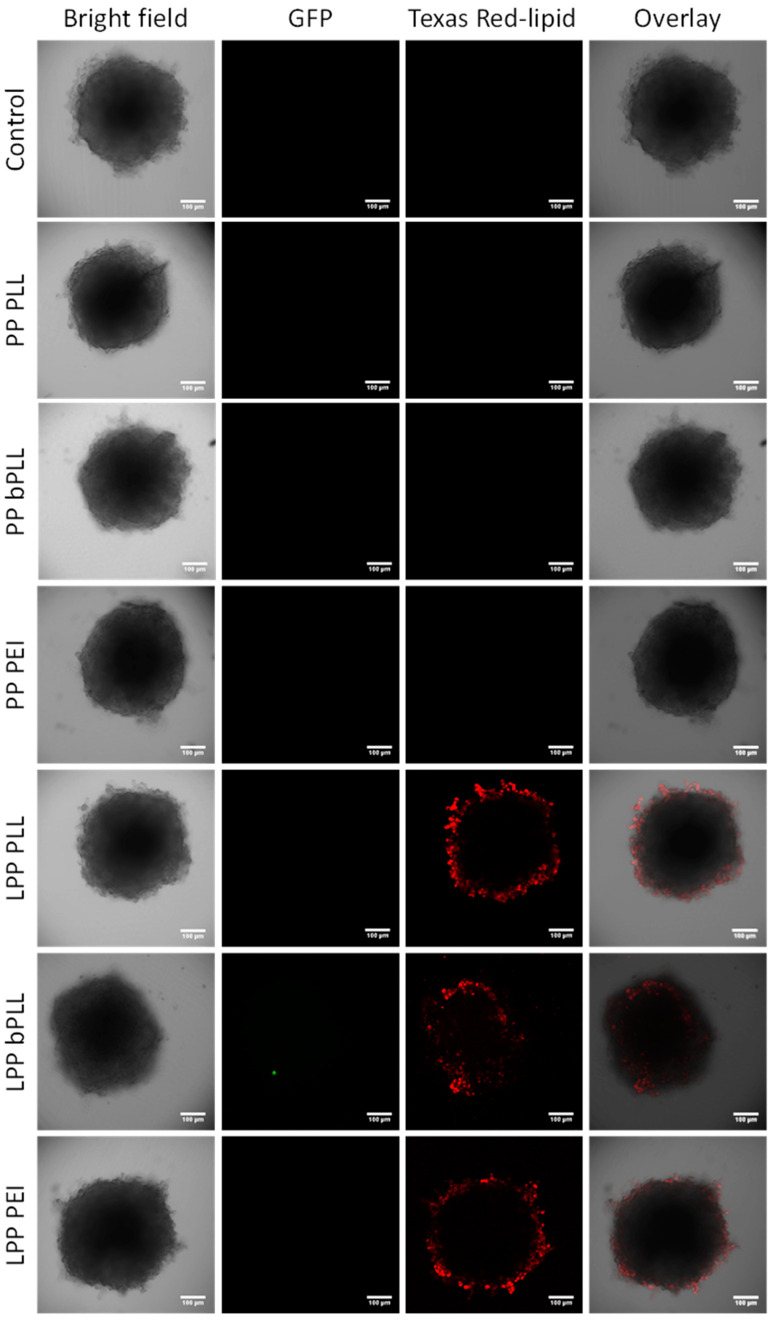
Confocal microscopy images of A549 cells in 3D multicellular spheroid culture 3 d after incubation with PPs and LPPs. The figure shows, from left to right, for each treatment: bright-field image, GFP fluorescence (green) as an indicator of transfection activity, Texas Red lipid fluorescence (red) reporting about the depth of penetration, and overlay of the three previous images.

The spheroids were then evaluated by confocal microscopy in order to assess two different parameters: GFP transfection with all the prepared formulations and penetration of LPPs in the multicellular spheroid. Regarding GFP transfection, no successful transfection was detected for any of the experimental groups (Figure 5). This result contrasted with the 2D monolayer cell culture, in which we had seen relatively successful transfection, at least for the cells incubated with LPP PEI. Even though previous works had shown some nonviral transfection in multicellular spheroids [35,45], this is to the best of our knowledge the first time that this type of LPP sample has been tested in this model, and many factors might have hindered gene transfection. For example, the penetration capacity of the formulation is a key parameter that can influence transfection efficiency. If the particles only interact with the outermost layer of cells in the spheroid, the number of cells that present effective nanoparticle uptake might be too small to observe significant GFP expression (for example, some GFP signal can be detected from only one cell in the LPP bPLL group in Figure 5). To evaluate the penetration capacity of the LPP formulations, confocal microscopy was used to monitor the presence of red fluorescence in the treated spheroids. Results (Figure 4B and Figure 5) show a very limited penetration capacity, with all the particles being retained on the spheroid surface (maximum penetration ca. 60 µm). The penetration capacity of LPPs in the context of cancer therapeutics would have a huge impact on their real clinical potential, so this 3D spheroid model would provide a more realistic initial evaluation for such applications. Despite the relatively good performance of some of the prepared formulations (mainly LPP PEI) in 2D monolayer culture, their poor penetration and transfection in this 3D spheroid model would, therefore, indicate that further work would be needed to enhance these parameters before considering moving to more therapeutically relevant plasmids or to a more complex biological model (such as in vivo evaluation).

### 2.4. Biological Evaluation in Microdroplet-Based Single-Cell Culture Model

**Figure 6:**
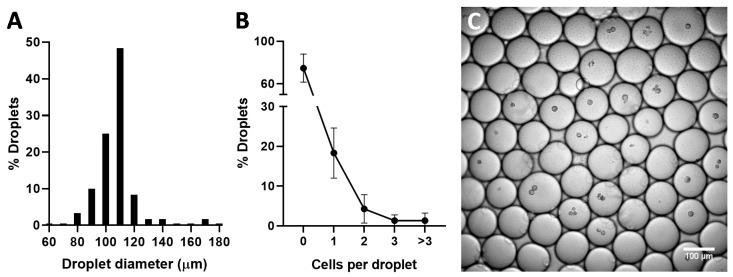
Histogram of microdroplet diameter (**A**); number of A549 cells encapsulated per microdroplet (*n* > 600) (**B**); example confocal microscopy image of obtained cell-containing microdroplets (**C**).

The third, and last, model tested consisted of encapsulating single A549 cells in microdroplets produced in a microfluidic device. This model has gathered much attention in recent years, and might be very useful to elucidate mechanistic aspects of the interaction of different types of gene delivery formulations with individual cells [40,46]. The characterization of the prepared droplets is shown in Figure 6. The prepared microdroplets presented an average diameter of 102.7 ± 13.2 µm, very close to the width of the microfluidic channels used to prepare them (100 µm). While the majority of the droplets were empty (74.7 ± 13.2%), 18.3 ± 6.3% contained one single cell, and fewer than 7% had two or more cells inside.

**Figure 7:**
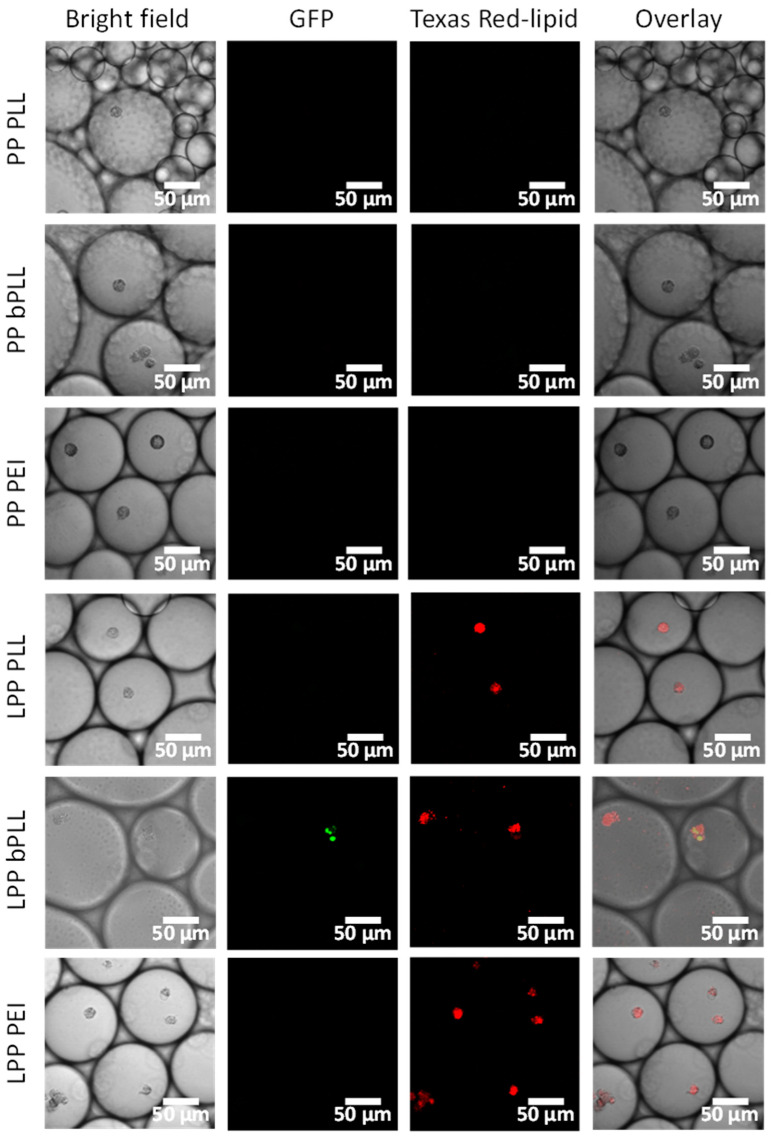
Confocal microscopy images of A549 cells in microdroplet-encapsulated cell culture 3 d after incubation with PPs and LPPs. The figure shows, from left to right, for each treatment: bright-field image, GFP fluorescence (green) for effective transfection, Texas Red lipid fluorescence (red) for successful internalization and overlay of the three previous images.

A549 cells were encapsulated in microdroplets together with the prepared PP and LPP formulations containing GFP-encoding plasmid. As was done for the previous two models, the cells were further incubated at 37 °C for three days and the cell-containing droplets were then evaluated by confocal microscopy (Figure 7). At least 300 droplets were imaged for each experimental group. The droplets were still present after incubation, and no drastic effect on droplet size was observed. In the LPP-treated groups, red fluorescence from the particles was observed in virtually all of the cells, indicating an extremely efficient cell uptake in this model. This positive result can be ascribed to the physical entrapment of the cells and formulations in confined droplets over all the experiments, maximizing the potential for interaction between cells and LPPs over the whole cell surface. Out of all of the tested conditions, GFP expression was only detected in the LPP bPLL treatment group (with 7.4% of the cell-containing droplets imaged showing green fluorescence). This result clearly contrasts with those obtained by 2D monolayer cell culture, where LPP PEI was the most efficient formulation for GFP transfection. Several considerations should be highlighted here to try to explain this difference in the behavior of the formulation across the different models. First, A549 cells are adherent cells, which were forced to remain in suspension for three days in this microdroplet-based model. This suboptimal culture conditions for this cell line might lead to a decreased expression of the introduced plasmids. On the other hand, particle uptake showed to be greatly enhanced in this model, so the transfection potential might be enhanced for formulations in which uptake or stability in the extracellular medium are the limiting factors. Out of the three LPP formulations prepared, LPP bPLL was expected to have the lowest stability, since it was designed to break upon reducing conditions. After uptake, this would lead to the release of very large amounts of plasmid, which could explain the behavior of this formulation as the best among the LPPs tested in this model.

In line with the other in vitro models, no GFP expression was observed for negatively charged PPs, even in these conditions that strongly favor cell uptake by forced confinement. These observations underline the importance of the positive surface charge on the particles.

Summarizing the results obtained with the three different models, we can observe that each model presents different advantages and disadvantages, being each one suitable for obtaining different insights about nonviral gene delivery formulations. This is highlighted by the fact that even comparing the same formulations in all three models, the performance of each formulation varied with the selected model, both in terms of toxicity and transfection efficiency. Two-dimensional (2D) and microdroplet-based cell cultures are more useful at earlier stages of research, for example when trying to optimize for endosomal escape or looking at endocytic trafficking. Selecting one or the other will depend on several factors. Two-dimensional (2D) cell culture is a simpler model that can be used for high-level live-cell imaging to gain intracellular mechanistic insights [14]. However, microdroplet-based culture allows for single-cell analysis that can provide different feedback from what is observed in 2D culture. As an example of single-cell analysis of nonviral gene delivery systems, we can highlight the recent work of Reiser et al. [47], where the authors presented live-cell imaging on a single-cell arrays (LISCA) platform. In that work, the fluorescence intensity of hundreds of transfected cells was monitored over time at the single-cell level, showing large variability among cells. These kinds of strategies could be combined with the phenotypic evaluation of each cell, looking for phenotype-efficacy correlations that could be used to optimize the formulations under evaluation. One advantage of the microdroplet-based culture would be the possibility of analyzing both adherent and suspension cells, while only adherent cells can be evaluated with the platform developed by Reisner et al. Another advantage of this system stems from the use of microfluidics, enabling high-throughput and automated analysis. Theoretically, one could make small modifications in the LPPs (or other types of formulations) and create a droplet array with all the compositional variables and evaluate all of them simultaneously to select the optimal one. This procedure can be repeated for different parameters, until all the relevant formulation characteristics are optimized. A limitation of this model in the context of adherent cells is that this setup is significantly different from the real-life environment of the cell (e.g., much more exposed area since the cells are in suspension instead of attached to a surface). This fact can however be exploited to probe specific steps in the delivery process, such as endosomal escape, since uptake will probably be enhanced in this model, preventing it from being the main limiting step. Another downside of microdroplet-based culture of adherent cells when compared with 2D culture is that live-cell imaging of the endocytic pathways within the microdroplets is more complex, given the mobility of the cells in the Z-axis caused by the cells being in suspension. In later stages of formulation development, and mainly in the context of solid tumors, 3D spheroid culture allows optimizing for other types of barriers since the tumor microenvironment can be recreated more accurately. On the other hand, 3D spheroids might not be suitable at all in the context of other diseases. A downside of the spheroid model is that it might not be suitable for initial formulation steps, since differences among formulations might not be observed unless some parameters have been previously optimized in simpler models. For example, in this work, no successful transfection was observed for any of the tested formulations in the 3D spheroid model, so no feedback could be obtained from this model regarding which one of these formulations might be worth developing further. From the results obtained in this work, we can reason that each model is suited to optimize different steps of formulation development for gene delivery. Given the complexity of cell-nanoparticle interactions, the conditions optimized for one model might not directly translate into other models, either in vitro or in vivo, so later reoptimization steps might be necessary. In any case, we believe that by understanding and acknowledging the capabilities and limitations of the different models that can be employed, the most suitable models for each particular step of a formulation project can be selected, which enables gaining as much information from it as possible, without misinterpreting the obtained data.

## 3. Materials and Methods

### 3.1. Reagents

Poly-l-lysine (PLL, 150–300 kDa, 1 mg/mL aqueous solution), Polyethyleneimine (PEI, branched, 25 kDa by LS), In Vitro Toxicology Assay Kit resazurin-based, Dulbecco’s Modified Eagle’s Medium-high glucose with glutamine (DMEM), penicillin–streptomycin (P-S) and fetal bovine serum (FBS) were purchased from Sigma Aldrich (Sigma Aldrich, Algés, Portugal). 1,2-dioleoyl-3-trimethylammonium-propane (DOTAP) and 1,2-dioleoyl-sn-glycero-3-phosphocholine (DOPC) were purchased from Avanti Lipids (Alabaster, AL, USA). Texas Red™ 1,2-Dihexadecanoyl-sn-Glycero-3-Phosphoethanolamine Triethylammonium Salt (Texas Red-DHPE) was purchased from Thermo Fisher (Fisher Scientific Lda., Porto Salvo, Portugal). GFP plasmid (pCMV-GFP), obtained through Addgene, was a gift from Connie Cepko (Addgene plasmid #11153) [48]. The peptide employed for the synthesis of bPLL (CKKKKKC) were purchased at POP-UP, Faculty of Sciences, University of Porto (Porto, Portugal).

### 3.2. Synthesis of Bioreducible Poly-l-Lysine (bPLL)

The bioreducible poly-l-lysine (bPLL) was obtained by oxidation of the CKKKKKC monomer (30 mg) by DMSO (200 µL) in PBS (700 µL) for 5 d, followed by purification by dialyzing against water at 4 °C (3.5 kDa cutoff) and freeze-drying. Successful preparation of the desired product was confirmed by ^1^H NMR [47] and Raman spectroscopy (confirming the formation of disulfide bonds, not present in the starting peptide [49,50,51,52,53,54]) (Figure S3).

### 3.3. Preparation of Polyplexes and Lipopolyplexes

Polyplexes (PPs) were prepared by mixing aqueous solutions of plasmidic DNA encoding GFP and cationic polymer (PLL, bPLL or PEI) polylysine at a certain charge ratio (CR, ratio between positive charges in the polymer to negative charges in the plasmid), and incubating them at room temperature for 30 min in an orbital shaker. For PP PLL and PP bPLL, CR = 1.5 was used, while a CR = 2 was selected for PP PEI. These CR values were selected to provide negatively charged PPs that could interact with cationic liposomes, but relatively close to CR values that would provide neutral particles (which would aggregate).

Texas Red-labeled cationic liposomes (Lipos, composition DOTAP/DOPC/Texas Red-DHPE 79.9/19.9/0.2) were prepared. First, the lipids were mixed in chloroform solution, followed by evaporation of the solvent, rehydration with water and sonication with a tip sonicator at a final total lipid concentration of 4 mM (sonication conditions: 10% amplitude, 1 min, 50% duty cycle using a Branson Digital Sonifier^®^ 250 Model) (Danbury, CT, USA).

Finally, both components (PP and Lipos) were mixed with a DOTAP nitrogen/DNA phosphate molar ratio of 0.75. A final DNA concentration of 46 µg/mL was fixed for all of the prepared samples. The obtained dispersions were characterized by Z Potential and DLS.

### 3.4. Characterization Techniques

^1^H NMR experiments were performed in a 400 MHz Bruker Avance II (Billerica, MA, USA) using D_2_O as a solvent. Raman spectroscopy measurements were performed in a 300R Confocal Raman microscope (WITec GmbH, Ulm, Germany) using a 785 nm laser line. The 10X objective was used, acquiring for 20 s per spectrum at a power of 100 mW. The different formulations obtained in this work were characterized by dynamic light scattering (DLS) and Z Potential using an SZ-100 device from Horiba (Kyoto, Japan). For each sample, 3 runs were performed with 10 measurements per run. Measurements were performed at 25 °C in deionized water employing a detector at an angle of 173°. TEM of the samples after negative staining with UranyLess© (following the manufacturer’s instructions) was carried out in a JEOL 2100 200 kV TEM (Izasa Scientific, Carnaxide, Portugal). Confocal microscopy was performed in a LSM780 equipment from Zeiss (Oberkochen, Germany). Images were taken using two different excitation laser lines: 488 nm for GFP and 561 nm for Texas Red-labeled lipid. A PMT detector (Zeiss, Oberkochen, Germany) was used to obtain transmission “bright field” images. Nonconfocal fluorescence microscopy was performed with a Nikon Eclipse Ti-E-Inverted fluorescence microscope (Izasa Scientific, Carnaxide, Portugal). Analysis of microscopy images was performed using ImageJ 1.52a Software. Flow cytometry was performed with a Bio-Rad S3e Cell Sorter from Bio-Rad (Hercules, CA, USA). GFP expression was evaluated from the signal in channel FL1 (excitation 488 nm laser line). Fluorescence spectroscopy for cell viability determination (through a resazurin assay) was measured in a Plate Reader SYNERGY H1 (Biotek) (Winooski, VT, USA), with a λ_exc_ = 550 nm and a λ_em_ = 595 nm.

### 3.5. 2D Monolayer Cell Culture

A549 cells (ATCC CCL-185) were seeded in 24-well plates at a density of 20,000 cells/cm^2^ 24 h before incubation with the nonviral gene delivery formulations. Then, 1 mL of PP or LPP suspension in DMEM (1 µg/mL DNA) were added per well and incubated at 37 °C, 5% CO_2_ and 95% humidity for 4 h. The medium was then replaced by fresh complete culture medium (DMEM with 10% FBS and 1% P-S), and the cells were incubated for another 72 h. The expression of GFP was evaluated by fluorescence microscopy and flow cytometry. Three replicates were performed per condition.

For the cytotoxicity evaluation of the formulations, a similar procedure was followed. After 24 or 72 h of incubation of A549 cells with the formulations (1 µg of DNA per well), a Resazurin-based In Vitro Toxicology Assay Kit was used following the manufacturer’s instructions and measuring the result by fluorescence spectroscopy. Three replicates were performed per condition.

### 3.6. 3D Multicellular Spheroid Culture

A549 cells were seeded at a concentration of 50,000 cells/mL of complete culture medium in a Nunclon Sphera 96U-well plate (Thermo Fisher, 200 µL per well) and incubated at 37 °C, 5% CO_2_ and 95% humidity for 3 d. Then, 100 µL of medium was carefully removed and replaced with fresh complete medium, and the spheroids were further incubated further for another 4 d. Then, 100 µL of medium was carefully removed and 100 µL of suspensions of the different formulations were added (1 µg of DNA per well), incubating the cells for another 72 h. Every 24 h, the spheroids were imaged with an inverted microscope to study spheroid growth. After 72 h, the spheroids were carefully transferred to a µ-Slide 18 Well #1.5 polymer coverslip (ibidi^®^) and imaged by confocal microscopy. Three replicates were performed per condition.

### 3.7. Microfluidic Devices Fabrication

**Figure 8:**
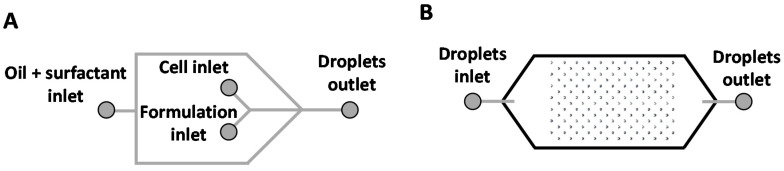
Schematic representation of the design of the microfluidic devices used in this work as microdroplet generators (**A**) and microdroplet reservoirs (**B**).

The microfluidic devices for microdroplet generation and incubation were fabricated by conventional photo- and soft lithography methods [55,56]. Briefly, the devices were designed with AutoCAD 2013 (Autodesk), and a dark-field mask was printed (JD-Photo Data, UK). A schematic representation of the device design can be seen in Figure 8.

For the microdroplet generators, SU-8 2025 negative photoresist (MicroChem) was spin-coated onto a silicon wafer (diameter: 76.2 mm) at 500 rpm for 5 s and at 1000 rpm for 33 s, aiming at a final thickness of 80 µm. A prebake before photolithography for 3 min at 65 °C and 9 min at 95 °C was performed. Then, the coated silicon wafer was exposed to UV light through the acetate mask on a mask aligner at 750 mJ cm^−2^ (MA6BA6, Suss Microtech). Afterwards, a post-bake was performed (1 min at 65 °C and 4 min at 95 °C) and, final development using SU-8 developer (PGMEA, Sigma Aldrich) was used to remove the nonpolymerized areas. The master was hard-baked for 2 min at 170 °C and profilometry (KLA-Tencor) showed a final thickness of 80 µm.

For the reservoirs employed for confocal imaging, SU-8 2025 negative photoresist was spin-coated onto a silicon wafer (diameter: 76.2 mm) at 500 rpm for 5 s and at 1450 rpm for 40 s. After spin-coating, the wafer was prebaked (2 min at 65 °C, then 6 min at 95 °C). The spin-coating and prebaking steps were repeated twice to obtain a final thickness close to 120 µm. After, the coated silicon wafer was exposed to UV light at 500 mJ cm^−2^, followed by a post-baking step (1 min at 65 °C and 3 min at 95 °C) and development in SU-8 developer. Finally, the master was hard-baked for 2 min at 170 °C and a final thickness of 120 µm was obtained, as measured by profilometry.

For the fabrication of the polydimethylsiloxane (PDMS, Sylgard 184) replicas, a mixture of PDMS prepolymer and cross-linker (ratio 10:1, *w*/*w*) was poured on top of the silicon master, degassed and cured for 2 h at 65 °C. The cured device was cut and peeled off from the master, and holes for tubing were made with a biopsy punch (diameter = 1 mm; Kai Medical). Finally, the PDMS piece containing the channels was either bonded to a standard glass slide (for the microdroplets generators) or to a cover slip (for reservoirs), following treatment with oxygen plasma for 30 s. Finally, to guarantee the optimal permanent bonding of all the small features within the device, soft pressure and a 10 min bake at 65 °C were applied. The channels of the droplet generator devices were functionalized by flushing Aquapel ^®^, followed by a fluorinated oil (HFE 7500 Novec) rinse to remove the remaining Aquapel ^®^ and to avoid crystallization which may derive in channel clogging. This step is crucial to improve the hydrophobicity of the channels to obtain monodisperse water in oil droplets.

### 3.8. Microdroplet-Encapsulated Cell Culture

The encapsulation of cells with the formulations (PPs and LPPs) was made using the microdroplet generators fabricated as described above. The microdroplet generators have one inlet for the continuous phase (oil), two inlets for the dispersed phase (cell and formulation inlets, both aqueous) and one outlet to recover the formed microdroplets (Figure 8). The devices with this T-junction design had a channel width of 100 µm and a height of 80 µm.

First, two syringes (1 mL, Terumo) were filled with the dispersed phases, one with 2 × 10^6^ A549 cells/mL and the other one with dispersions of the formulations at a DNA concentration of 9.2 µg/mL, both in complete culture medium. Another syringe (1 mL, Terumo) was filled with the continuous phase (HFE 7500, Novec, + Pico-Surf 1–2%, Sphere Fluidics, Ltd., Cambridge, UK). The syringes were mounted on syringe pumps (New Era Pump Systems), and the tubing ends were connected to the microfluidic devices. The syringe pumps were programmed for specific flow rates for the oil (Q_continuous_ solution) of 1000 µL/h and the aqueous dispersions (Q_aqueous_ dispersions) of 500 µL/h.

Then, the generated microdroplets were recovered through the outlet channel using an additional tubing piece, and transferred into 1 mL syringes previously filled with 100 µL of HFE 7500 + Pico-Surf 1 (2%) solution. Once enough microdroplets were collected in the syringe (≈ 400 µL), the system was closed using a flat-end tubing for storage in the incubator (hot tweezers were used to close the end of the tubing). The resultant syringe with the microdroplets-containing A549 cells and formulations were kept in an incubator at 37 °C, 5% CO_2_ and 95% humidity for 72 h. Then, they were transferred to a microdroplet reservoir by opening the flat-end tubing attached to the storage syringe, connecting it to the reservoir and gently injecting a small amount of droplets to be imaged by confocal microscopy.

## 4. Conclusions

Polyplex and lipopolyplex formulations based on three different cationic polymers were prepared, characterized and evaluated in three different in vitro culture models: 2D monolayer cell culture, 3D multicellular spheroid culture and microdroplet-based single-cell culture. The prepared polyplexes with low charge ratios showed negligible transfection across all three tested models and, while 2D cytotoxicity data showed no deleterious effect on cell viability, these formulations (and especially PP PLL) were shown to hinder 3D multicellular spheroid growth. Regarding the biological evaluation of lipopolyplexes, great differences were observed based on the in vitro model employed. All lipopolyplex formulations were well tolerated and did not produce any measurable cytotoxic effects neither in 2D nor in 3D cell culture. In 2D monolayer cell culture, the formulation LPP PEI performed the best, showing significant GFP transfection, even though LPP bPLL also showed some transfection potential. In 3D spheroid culture, none of the tested formulations presented successful GFP transfection, possibly due to the extremely limited penetration capacity of the formulations in the model, which remained in contact only with the outer layer of cells. Finally, in the microdroplet-encapsulated cell culture model, the formulation LPP bPLL was the only one that showed transfection capacity, possibly due to the large release of GFP plasmid after uptake, which was observed to be extremely efficient in this model. Taken together, these results show that, at least in the context of lipopolyplexes, care should be taken when comparing results from different in vitro models, and more consideration should be put into in vitro experimental planning.

## Figures and Tables

**Table 1:** Characterization of the prepared particles by DLS and Z Potential.

Sample	Hydrodynamic Diameter (nm)	PDI	Z Potential (mV)
PP PLL	115.4 ± 0.7	0.26 ± 0.03	−49.5 ± 2.1
PP bPLL	87 ± 19	0.68 ± 0.38	−39.3 ± 5.5
PP PEI	138.7 ± 2.8	0.43 ± 0.05	−38.1 ± 0.9
Lipo	112.2 ± 1.3	0.33 ± 0.03	+99.5 ± 2.1
LPP PLL	121.8 ± 0.9	0.21 ± 0.06	+47.5 ± 0.7
LPP bPLL	168.1 ± 6.2	0.38 ± 0.02	−11 ± 0.8
LPP PEI	149.1 ± 21.5	0.33 ± 0.06	+35.9 ± 1.7

## Supplementary Materials

Figure S1: Negative-stained TEM micrographs of PP PLL, Lipos and LPP PLL. Figure S2. Resazurin viability assay of A549 cells incubation with PPs and LPPs for 72 h. Figure S3. ^1^H NMR and Raman spectra of the peptide CKKKKKC and the prepared bioreducible polymer bPLL.
