# Supplementary material for: In Vitro Evaluation of Lipopolyplexes for Gene Transfection: Comparing 2D, 3D and Microdroplet-Enabled Cell Culture
Source: Molecules. 2020 Jul 18;25(14):3277. doi: 10.3390/molecules25143277 (PMC7397275; doi:10.3390/molecules25143277)
Supplement: Supplementary file 1 [file molecules-25-03277-s001.pdf]

SUPPORTING INFORMATION

In vitro evaluation of lipopolyplexes for gene transfection: comparing 2D, 3D and microdroplet-enabled cell culture

Juan L. Paris, Filipe Coelho, Alexandra Teixeira, Lorena Diéguez, Bruno F.B. Silva, Sara Abalde-Cela.

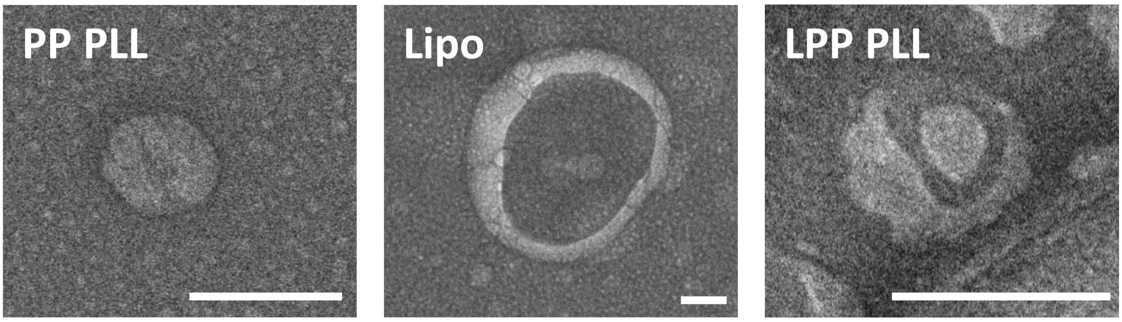

Figure S1. Negative-stained TEM micrographs of PP PLL (left), Lipos (center) and LPP PLL (right). Scale bars represent 100 nm.

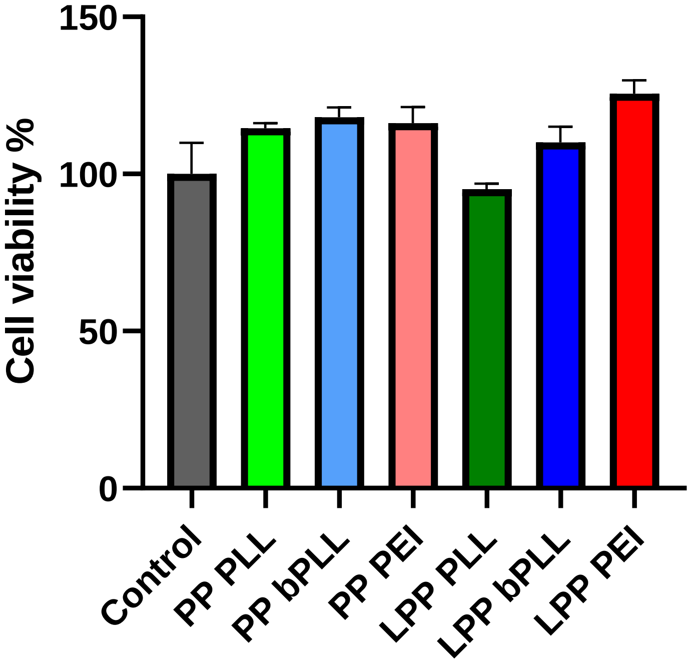

Figure S2. Resazurin viability assay of A549 cells incubation with PPs and LPPs for 72 h. Data are Means ± SD, n=3.

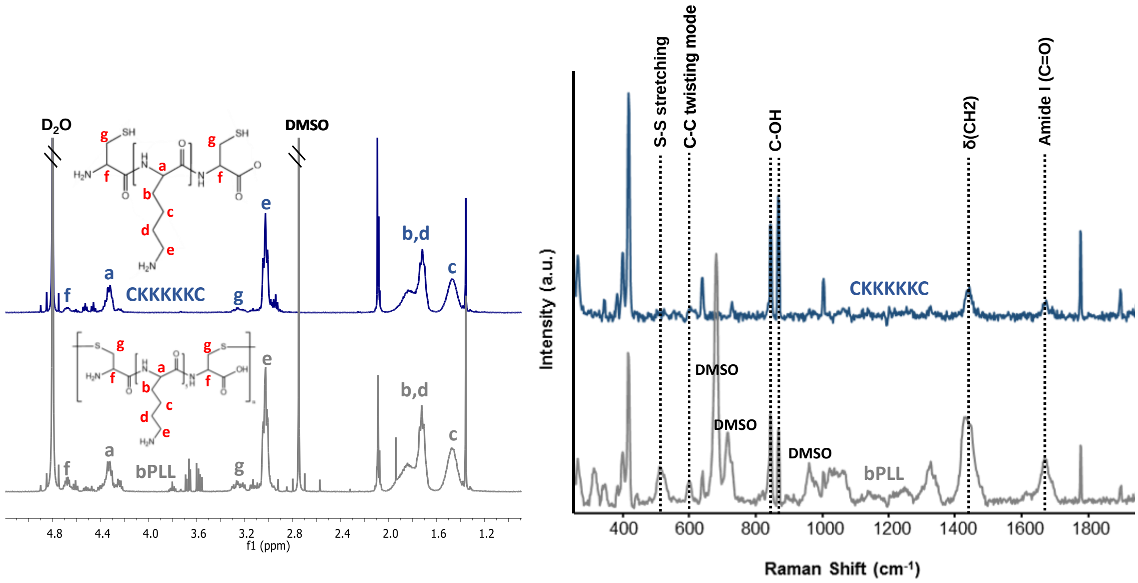

Figure S3. ^{1}H NMR (left) and Raman (right) spectra of the peptide CKKKKKC (blue) and the prepared bioreducible polymer bPLL (grey).
